# Critical-Illness: Combined Effects of Colistin and Vasoactive Drugs: A Pilot Study

**DOI:** 10.3390/antibiotics12061057

**Published:** 2023-06-15

**Authors:** Rodopi Stamatiou, Anna Vasilaki, Dimitra Tzini, Vasiliki Tsolaki, Konstantina Zacharouli, Maria Ioannou, George Fotakopoulos, Markos Sgantzos, Demosthenes Makris

**Affiliations:** 1 Physiology Laboratory, Faculty of Medicine, University of Thessaly, Biopolis, 41500 Larissa, Greece; 2 Laboratory of Pharmacology, Faculty of Medicine, School of Health Sciences, University of Thessaly, Biopolis, 41221 Larissa, Greece; 3 Intensive Care Unit, Faculty of Medicine, University of Thessaly, Biopolis, 41500 Larissa, Greecedimomakris@uth.gr (D.M.); 4 Pathology Department, Faculty of Medicine, University of Thessaly, Biopolis, 41500 Larissa, Greece; 5 Department of Neurosurgery, University Hospital of Larissa, 41500 Larisa, Greece; 6 Anatomy Department, Faculty of Medicine, University of Thessaly, Biopolis, 41500 Larissa, Greece

**Keywords:** colistin, sepsis, myopathy, vasoconstrictive drugs, critical illness

## Abstract

Colistin is often used as a last resort for treating multidrug-resistant infections, particularly in critically ill patients in intensive care units. Nonetheless, its side effects, including myopathy, require careful monitoring. Vasoconstrictive drugs are also used in intensive care to increase blood pressure and improve blood flow to vital organs, which can be compromised in critically ill patients. The exact mechanism of colistin-induced muscle toxicity is of significant interest due to its potential intensive-care clinical implications. Colistin alone or in combination with vasoconstrictive agents was administrated in non-septic and LPS-induced septic animals for 10 days. Histopathological evaluation of the gastrocnemius muscle and dot-blot protein tissue analysis were performed. Increased intramuscular area, de-organization of the muscle fibers and signs of myopathy were observed in colistin-treated animals. This effect was ameliorated in the presence of vasoconstrictive drugs. Administration of colistin to septic animals resulted in a decrease of AMPK and cyclin-D1 levels, while it had no effect on caspase 3 levels. Vasoconstrictive drugs’ administration reversed the effects of colistin on AMPK and cyclin D1 levels. Colistin’s effects on muscle depend on septic state and vasoconstriction presence, highlighting the need to consider these factors when administering it in critically ill patients.

## 1. Introduction

Bacterial antimicrobial resistance (AMR) has emerged as one of the leading public health threats of the 21st century, as indicated by the 4.95 million deaths (3.62–6.57) per year associated with it [1]. AMR has been documented in both Gram-positive and -negative bacteria leading to an increasing need for the development of new antibiotics and allocate existing drugs more effectively [2]. One of the factors that have contributed to AMR is the lack of organized health protocols that will provide healthcare professionals with the ability to characterize the pathogen that caused the infection as soon as possible and therefore prescribe the most effective antibiotic. Therefore, initial treatment is conducted by broad-spectrum antibiotics. However, the use of such drugs is regarded to be a factor that can lead to evolutionary changes in bacteria that can result in AMR [3]. Physicians face limitations regarding the pharmacological agents available for treating AMR. According to the 2019 Center for Disease Control (CDC) Antibiotic Resistance Threats Report, more than 35,000 people die in the USA every year of antibiotic-resistant infections, while the number of patients infected is over 2.8 million people [4]. Similar studies from European countries show similar results, estimating that over 10 million people will face AMR every year worldwide, while the cost in the world economy will raise to $100 trillion by 2050 [5]. In many cases, multiple combinations of different antimicrobial agents are necessary, and even then, their effectiveness can vary. The concurrent administration of multiple antibiotic agents can increase their pharmacodynamic killing activity and potentially suppress or delay the emergence of resistance by broadening the spectrum of activity and exploiting different mechanisms of action [2,3,4]. Even though AMR can be described for any bacterium, the most common bacteria that present antibiotic treatment resistance are *Enterococcus faecium*, *Staphylococcus aureus*, *Klebsiella pneumoniae*, *Acinetobacter baumannii*, *Pseudomonas aeruginosa*, *Escherichia coli* and *Enterobacter* species, characterized by the acronym ESKAPE/ESCAPE [6,7,8,9]. The need for effective drug combinations is, more than ever, eminent so as to prevent the spread of antibiotic resistance to other bacterial species as well [8]. Efforts have been made in order to identify the molecular interactions between antibiotics, such as carbapenems, and various bacterial components, like the *Acinetobacter baumannii* oxacillinases, aiming to propose effective antibiotic treatment [10]. These studies have used molecular docking analysis to provide new insights regarding the effectiveness of antibiotics based on their interaction with bacterial enzymes [10]. 

Colistin is an antibiotic of the polymyxin B class that consists of antibiotics effective against mainly Gram-negative bacteria. These antibiotics are cyclic peptides with hydrophobic tails that have a positive charge and were originally found as products of the *Paenibacillus polymyxa*, a Gram-positive bacterium [11]. The way of action polymyxins is through disruption of the balance between polar and hydrophobic properties of the membrane, therefore altering the permeability of the microorganisms [12,13]. Colistin has been used as a veterinary drug for the treatment of domestic and productive animals, especially against *Enterobacteriaceae*, both for prophylaxis and therapy [13]. It has also been used extensively as a growth promoter in animals, raising questions about the safety of people from the presence and evolutionary prevalence of colistin-resistant microbes, which has led to restrictions on the use of polymyxins [13]. Namely, the use of antibiotics in animal growth can reduce the number of symbiotic bacteria in the digestive system of animals treated with antibiotics and, therefore, increase the weight of animals. However, the presence of these drugs in the animal tissues or animal products that are to be used as food leads to human exposure to the antibiotic through the food chain. Therefore, the safety of this specific way of antibiotic use in animal husbandry is in question regarding the resistance that can raise in bacterial species.

In medical practice, colistin has been used as a last-resort agent for the treatment of infections due to Gram-negative bacteria with difficult-to-treat resistance, especially carbapenem-resistant *Acinetobacter baumannii*, carbapenem-resistant *Enterobacterales*, and carbapenem-resistant *Pseudomonas aeruginosa* [14,15,16,17]. These pathogens are often identified as bacteria that infect hospitalized patients that already have health problems and can therefore be an additional burden on their clinical condition. Yet, colistin administration is accompanied by certain side effects that can be presented both in short- and long-term administration, as well. Among them, the most commonly recognized are: nephrotoxicity in the form of acute renal failure, acute interstitial nephritis, and tubular concentration defects are the most common adverse effects of colistin and neurotoxicity. The most frequently experienced neurological adverse effects are paresthesia, tingling sensations and autonomic instability, and are reported by approximately 27% and 7.3% of patients receiving intravenous (IV) and intramuscular colistin methate sodium, respectively [18]. Very rarely, colistin may cause muscle weakness secondary to reduced Acetylcholine (Ach) release with or without postsynaptic blockade of the Ach receptor, affecting the neuromuscular junction and therefore reducing muscle contractility [19]. The exact mechanism of muscle toxicity is not known but is attributed to a presynaptic action of colistin that interferes with the receptor site and blocks the release of acetylcholine to the synaptic gap [19,20]. Other studies reveal that treatment with colistin can also affect ICU patients hemodynamically [20] or even cause rhabdomyolysis [21]. 

Apart from these findings, critical myopathy/neuromyopathy is a common entity, affecting 30–70% of ICU patients and presenting during the early stages of critical illness [22,23,24,25,26]. Recognized risk factors are sepsis, immobilization and inadequate feeding, factors commonly presented in such patients with AMR as well. Therefore, the potential worsening of myopathy due to the administration of colistin may further complicate risk-benefit weighting before starting colistin. The use of corticosteroids has also been identified as a risk factor, while the role of neuromuscular blockers in the development of myopathy is still controversial [25,27]. Recently, in a cohort of mechanically ventilated critically-ill patients, vasoactive drugs were found to be independently associated with ICU-acquired weakness (AW) at hospital discharge [26]. According to this study, the duration of vasopressor support and the cumulative dose of the β-agonist norepinephrine significantly increased the odds of developing ICU-AW. Importantly, this effect was independent of other known risk factors for ICU-AW, including sepsis, markers of severity of illness, such as APACHE II score, and duration of mechanical ventilation. Moreover, norepinephrine and not vasopressin were independently associated with ICU-acquired weakness, suggesting that there are various signaling pathways that are involved in their action and can affect the induction or the progression of AW or even the interaction with other used drugs [27].

Several studies have focused on the elucidation of the molecular mechanisms activated during sepsis in both animal models and patients. Adenosine monophosphate (AMP)-activated protein kinase (AMPK) has been found to be activated in septic tissues and to been linked to a protective mechanism [28]. This mechanism leads to the activation of signaling pathways involved in metabolism reprogramming in order to preserve energy balance, even though the AMPK activation can lead to organ dysfunction [29]. Regarding colistin, it has been found to induce autophagy and apoptosis in cell cultures by activating the p53 pathway [30]. The autophagy pathway can be either a way of cell survival or a precursor of cell death via apoptotic caspase pathway activation [31]. Furthermore, sepsis has been found to be associated with G1 cell cycle arrest and cyclin D downregulation after p53 pathway activation [32]. 

The aim of the present study was to identify the possible negative impact of colistin therapy concerning myopathy in an LPS-induced septic model, simulating the sepsis that mechanically ventilated patients can develop. The additional effect of the combination of vasoactive drugs (norepinephrine or vasopressin) was assessed as well. 

## 2. Results

### 2.1. Animal Observations

**Figure 1:**
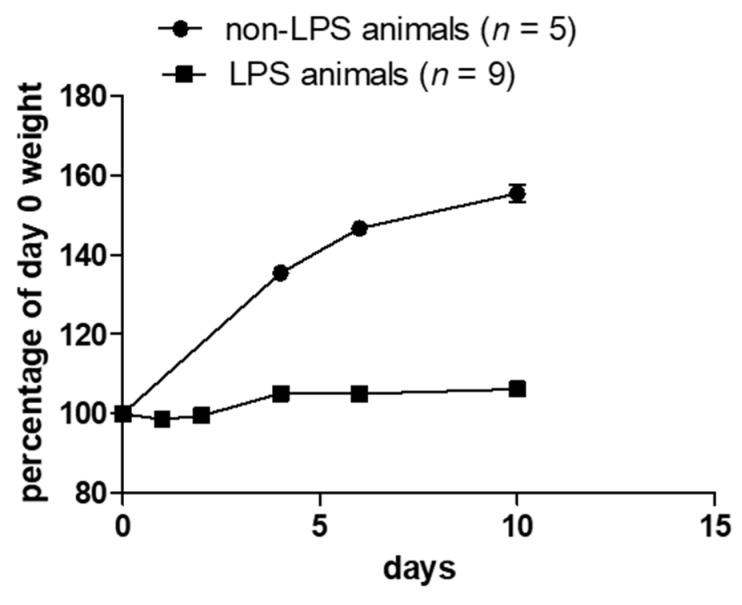
Weight measurement in non-LPS and LPS animals. LPS animals do not have the same growth rate as non-LPS animals.

There were no specific differences observed in septic and non-septic animals regarding motility, appetite or behavior. The only observed effect of sepsis was the presence of watery feces and the reduction in growth rate by 40%, as assessed by weight gain measurement (Figure 1). Enlarged and inflamed bowel was also observed in the LPS-animals group at the post-mortem examination during the tissue harvest. 

### 2.2. Histopathological Evaluations

**Figure 2:**
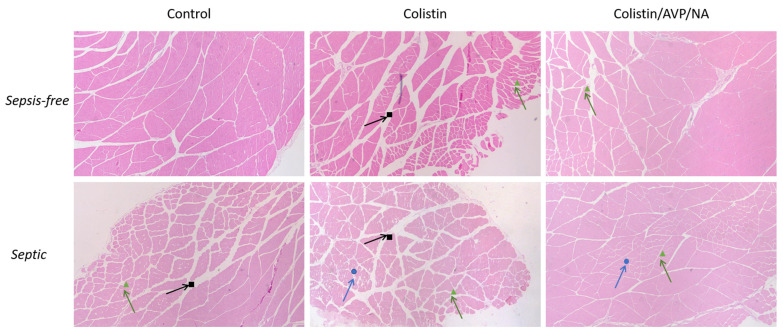
Effect of Colistin and Colistin/AVP/NA treatment on the gastrocnemius muscle of sepsis-free and septic animals: histopathological analysis of the muscle specimens. Signs indicate the areas of interest. Black squares show increased intramuscular area. Green triangles show the triangulation of muscle fibers. Blue circles show fiber degradation. AVP: arginine vasopressin, NA: noradrenaline. H&E staining, 40× magnification.

LPS-induced sepsis led to the enlargement of the intramuscular area, with a more triangular muscle fiber structure also observed regardless of the presence of colistin (Figure 2). The presence of colistin led to additional muscle deterioration, which had the characteristics of neurogenic myopathies, such as atrophy, cell death and de-organization of the muscle fibers. Additionally, areas with hyalinization were present in muscle specimens from animals treated with colistin alone. Conversely, the presence of vasoconstrictive drugs in combination with colistin in both non-septic and septic animals seems to ameliorate the colistin-induced effect without being able to completely inhibit any other effect (Figure 2).

To summarize, the histopathological evaluation of the muscle revealed that sepsis induces abnormalities in muscle structure, with colistin further exacerbating this effect and inducing further muscle destruction. However, the effect of colistin seems to be ameliorated in the presence of vasoconstrictive drugs, regardless of the state the animal is in, namely septic or sepsis-free.

### 2.3. Protein Analysis

**Figure 3:**
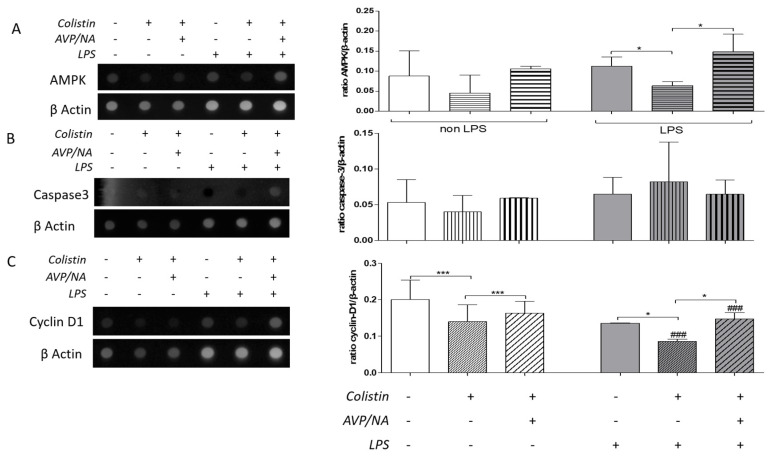
Effect of Colistin and Colistin/AVP/NA treatment on (**A**) AMPK, (**B**) caspase-3, and (**C**) cyclin D1 protein levels in the gastrocnemius muscle of sepsis-free and septic animals. (*): designates statistically significant differences in a pairwise comparison as indicated by the connective line, while (#) designates statistically significant differences between control and LPS-treated animals that have received the same drug treatment. * *p* < 0.05, *** *p* < 0.001, ^###^ *p* < 0.001 using the Mann–Whitney test. AVP: arginine vasopressin, NA: noradrenaline, AMPK: adenosine monophosphate (AMP)-activated protein kinase.

Colistin alone or in combination with AVP and NA had no effect on AMPK skeletal muscle levels of sepsis-free animals. In septic animals, colistin treatment led to a decrease in AMPK levels (* *p* < 0.05, LPS-treated animals vs. LPS- and colistin-treated animals), while the presence of vasoconstriction seems to block the colistin effect (* *p* < 0.05, LPS- and colistin-treated animals vs. LPS- and colistin/AVP/NA-treated animals; Figure 3A).

Skeletal muscle caspase-3 levels were not significantly affected by either sepsis or drug treatment (Figure 3B), as estimated by the dot-blot of total protein from muscle homogenates.

On the other hand, colistin decreased skeletal muscle cyclin D1 levels both in sepsis-free (*** *p* < 0.001, colistin-treated vs. control animals) and septic animals (* *p*< 0.05, LPS-treated vs. LPS- and colistin-treated animals; Figure 3D). The effect of colistin on cyclin D1 levels was more pronounced in septic animals compared to sepsis-free ones (### *p*< 0.001, colistin-treated vs. LPS- and colistin-treated animals). Vasoconstriction elevated colistin effects on cyclin D1 levels in both sepsis-free (*** *p*< 0.001, colistin-treated vs. colistin/AVP/NA-treated animals) and septic animals (*** *p* < 0.001, LPS- and colistin-treated vs. LPS-and colistin/AVP/NA-treated animals). This effect was more pronounced in septic animals (### *p*< 0.001, colistin/AVP/NA- treated animals vs. LPS- and colistin/AVP/NA-treated animals; Figure 3C).

Therefore, both sepsis and colistin alter the metabolic balance and reduce cell proliferation in rat skeletal muscle but do not seem to induce cell death, as estimated with caspase-3 level evaluation. Consistent with the histopathological observations, vasoconstriction reversed the effects of colistin on both AMPK and cyclin D1 levels.

### 2.4. Colistin Molecular Target Prediction and Docking

Upon searching the ChEMBL database using the term “*colistimethate*” and its alternative names, we obtained a list of compounds with identical target prediction profiles, such as CHEMBL2304327. On the other hand, the utilization of the SwissTargetPrediction web tool for target prediction of colistin resulted in the following prediction profile: Colistin_*Rattus Norvegicus*. Upon examining the profiles provided, acetylcholinesterase emerged as a potential colistin target associated with skeletal muscle and/or the neuromuscular junction function. 

**Figure 4:**
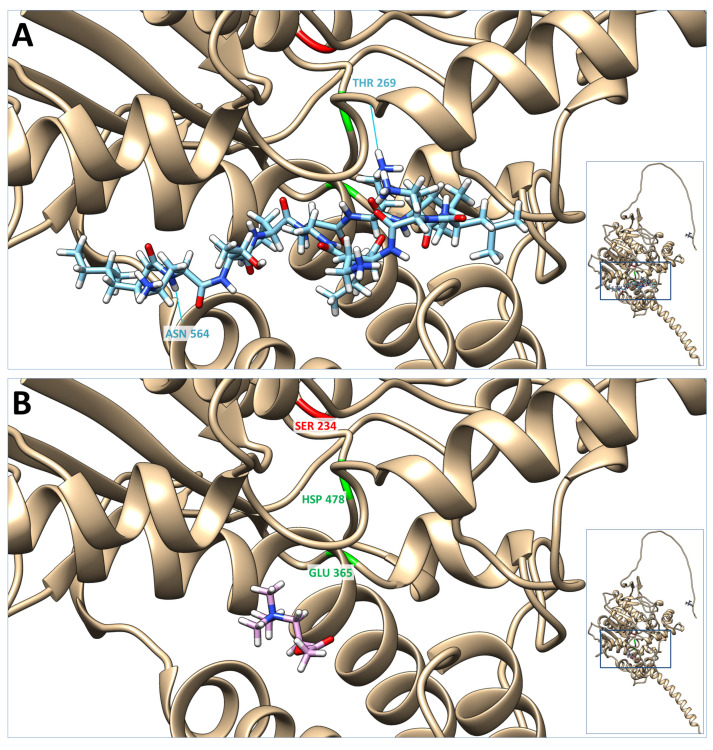
Molecular docking of colistin and acetylcholine on the acetylcholinesterase structure UniProt_P37136; AlfaFold predicted structure (inserts). Presented here are the lowest energy poses of colistin (**A**) and acetylcholine (**B**). In red is the acyl-ester intermediate active site (SER 234), and in green is the charge-relay system (GLU 365 and HSP 478) of the acetylcholinesterase active site. Blue lines represent the hydrogen bonds between colistin and the acetylcholinesterase molecule (i.e., #1.1 LIG 1 H74-#O ASN 564 O 1.947 Å and #1.1 LIG 1 NH1-THR 269 O 1.947 Å).

To further explore the possibility of colistin-acetylcholinesterase interaction, we conducted a molecular docking simulation using SwissDock and compared it with that of acetylcholine (Figure 4). Our findings reveal that the cluster with viable poses of colistin, which exhibited the lower energy values and the largest number of poses, indicative of more stable and favorable binding interactions, had DeltaG values ranging from −10.561111 to −10.267738. Meanwhile, the DeltaG values for acetylcholine ranged from −7.3974614 to −6.726739. These results suggest that colistin has a potentially stronger binding affinity and more favorable binding interaction with acetylcholinesterase compared to acetylcholine which supports the possibility of colistin exhibiting a higher affinity for acetylcholinesterase than acetylcholine.

## 3. Discussion

LPS treatment negatively affects skeletal muscle morphology. Namely, the intramuscular area increases, and the fibers appear to be fragmented and triangular in shape rather than an undivided tissue with round muscle fibers. These findings are in agreement with mixed myopathy with neurogenic characteristics [23]. The presence of colistin appears to aggravate LPS-induced effects, including the appearance of necrotic/apoptotic areas in the specimens (Figure 2). However, colistin’s effects were less pronounced when the animals were treated with vasoconstrictive agents. It is well known that vasoconstriction can affect the effects of drugs on tissues by reducing their delivery, elimination, absorption, and distribution within the tissues. Though reduced tissue elimination could lead to the accumulation of colistin in skeletal muscles, which in turn could lead to an increase in its effect, the concomitant administration of colistin with vasoconstrictive agents probably reduces its skeletal muscle delivery, absorption and distribution, which possibly causes an overall decrease in the effects of the drug on the tissue.

In sepsis-free animals, colistin alone or in combination with vasopressin and noradrenaline had no statistically significant effect on the levels of AMPK and caspase-3, while it decreased the levels of cyclin D1. This suggests that colistin may have a specific effect on the regulation of cyclin D1 in skeletal muscle, which may be independent of the presence or absence of vasoconstrictive drugs.

In septic animals, colistin treatment led to a decrease in the levels of AMPK, suggesting that the septic state may alter the effect of colistin on AMPK regulation. One of the key features of sepsis is the dysregulation of the body’s metabolism, which can lead to a state of energy deprivation. In order to counteract this energy deficit, the body employs several mechanisms to shift cellular metabolism toward energy conservation. One such mechanism involves the activation of AMPK, which is a key regulator of cellular energy homeostasis. AMPK is activated in response to a variety of stresses that deplete cellular energy stores, such as nutrient deprivation, hypoxia, and oxidative stress. In sepsis, the recruitment of AMPK helps to shift cellular metabolism towards the saving of energy by inhibiting energy-consuming processes such as protein synthesis and promoting energy-conserving processes such as autophagy and mitochondrial biogenesis [28,33]. This shift in cellular metabolism is critical for the survival of cells during periods of energy deprivation and can help to prevent cell death and tissue damage. Overall, the recruitment of AMPK in sepsis plays a crucial role in maintaining cellular energy homeostasis and promoting cell survival in the face of metabolic stress. The impact of colistin on sepsis may be linked to a reduction in AMPK activity, which has been found to result in the abnormal buildup of autophagy-related proteins and impaired mitophagy, thus bridging cellular energy sensing with autophagy and mitophagy [28,31,34].

Interestingly, the presence of vasoconstriction seemed to block the effect of colistin on AMPK levels, indicating that vasoconstriction may have a protective effect on the septic skeletal muscle response to colistin. This vasoconstriction effect is probably mediated through decreased skeletal muscle colistin delivery, absorption and/or distribution. It is, after all, known that in sepsis, the recruiting of AMPK helps the cell’s metabolism shift towards the saving of energy.

Caspase-3 levels were not significantly affected by either sepsis or drug treatment, suggesting that colistin and vasoconstriction may not have direct effects on skeletal muscle apoptosis in sepsis. However, it is important to note that apoptosis is a complex process that involves multiple signaling pathways, and other apoptotic markers or pathways may be affected by colistin or vasoconstriction in skeletal muscle. Furthermore, even if colistin has been reported to induce apoptosis via p53 activation in cell models [30], the effect in tissues can be more complex. Studies have shown that even though the role of caspase-3 is detrimental in the apoptosis pathway, there are different binding sites that can be occupied by different components and, therefore, can increase the complexity of this signaling pathway [35].

The most striking finding of this study was the effect of colistin on cyclin D1 levels, which decreased in both sepsis-free and septic animals, with a more pronounced effect in septic animals. This finding suggests that colistin may have a role in modulating cell cycle regulation in skeletal muscle. Cyclin D1 is a key regulator of the G1-S transition in the cell cycle, and a decrease in cyclin D1 levels may contribute to impaired muscle regeneration and repair. On the other hand, both colistin and sepsis itself have been shown to lead to G1 arrest, as cells attempt to minimize energy consumption and protect themselves from dying [32,34,36]. The observation that vasoconstriction elevates the effects of colistin on cyclin D1 levels suggests that vasoconstriction may decrease the skeletal muscle bioavailability or potency of colistin.

Even though the number of animals used in the present study was a limitation that, most possibly, affected the statistical significance of the presented results, overall, our results suggest that the effects of colistin on skeletal muscle protein levels may be influenced by the septic state and the presence or absence of vasoconstriction. These findings highlight the importance of considering these factors when administering colistin to critically ill patients.

In an attempt to further investigate the effects of colistin on skeletal muscle physiology, we performed a molecular docking simulation to the neuromuscular junction ChEMBL predicted a possible target of colistin, acetylcholinesterase. Based on our results, colistin has a potentially stronger binding affinity and more favorable binding interaction with acetylcholinesterase compared to acetylcholine. If colistin indeed interacts with acetylcholinesterase and affects its activity, it could disrupt the delicate balance of acetylcholine signaling, leading to muscle weakness, impaired muscle coordination, or other neuromuscular complications. Understanding the significance of this interaction is crucial to assess potential adverse effects associated with colistin administration.

Further studies are needed to clarify the underlying mechanisms and implications of these effects fully. It is also important to investigate the clinical relevance of these findings and how they could be used to optimize the dosing and administration of colistin to critically ill patients. By gaining a better understanding of how colistin and vasoconstrictive drugs affect skeletal muscles, we can develop more effective treatment strategies for sepsis and other critical illnesses.

## 4. Materials and Methods

### 4.1. Animals 

Fourteen immunocompetent Wistar rats were used. Animals were housed in regulation boxes and given free access to food and water while having a 12 h day-night circle. This study was carried out in strict accordance with the recommendations in the Guide for the Care and Use of Laboratory Animals [37], as well as institutional guidelines. All animals were male adults, 6–8 weeks old. All rats were able to move freely in spacious cages and were acclimatized by the researchers that were going to do both the daily treatment and the tissue harvesting after euthanasia. The animals appeared to have normal development, behavior and interactions with each other. No more than 3 animals were maintained in the same cage, while the room temperature varied from 20 to 25 °C, with ventilation and in a quiet animal house, away from disturbances.

### 4.2. Animal Treatment 

Five animals were sepsis-free, while 9 were LPS-induced septic animals. The septic animals received 250 mg/kg LPS (Lipopolysaccharides from Escherichia coli, 10,000 EU/mg, purified by phenol extraction, Sigma-Aldrich, Merck KGaA, Darmstadt, Germany) 5 days prior to any other drug treatment administration [38]. LPS was injected intraperitoneally. Sepsis was confirmed by a reduction in the weight-gain rate (Figure 1). Both the sepsis-free and the septic animals were weighed every day, 5 days prior to any drug administration and during the treatment and measurements were recorded. The rats were further randomly ascribed to 3 drug treatment groups; control animals, colistin-treated animals and animals treated with colistin and vasoconstrictive drugs. Control animals received sham injections of normal saline solution, colistin-treated animals received colistin (150,000 U/kg/day; COLISTIN/NORMA^®^, colistin methate Sodium, 1,000,000 IU/vial, Norma), and colistin and vasoconstrictive-treated animals received colistin (same as before) combined to arginine vasopressin (AVP: 4 U/kg/day; Empressin^®^; one mg is equivalent to 530 units according to the USA Food and Drug Administration) and/or nor-adrenaline (NA: 25 μg/kg/day). All drugs were administered intraperitoneal (IP) once/day for 10 consecutive days. During the treatment, animal behavior, movement, appetite, and feces production were monitored. 

### 4.3. Tissues Processing

Each animal was euthanized by decapitation, and the right gastrocnemius muscle was collected. Specimens were used for both histopathologic examination after Hematoxylin-eosin staining and protein level analysis using anti-AMPK, anti-caspase-3, anti-cyclin D1 and anti-β-actin antibodies. Any excessive tissue was carefully removed from muscles, while any visible excess neighboring tissue or fat was also removed from the specimen. Every tissue was divided into 2 equal parts by athwart cutting the muscle with a scalpel in the mid. One of these parts was used for protein analysis, while the other was for histopathological evaluation. The tissue part that was going to be histopathologically evaluated was embedded in a fixing agent, namely 4% formalin and placed in a transfer vial filled with formalin. The specimen stayed in formalin for more than 48 h prior to any further processing. The other half of the tissue was placed in a vial that was suitable for −80 °C storage, without any fixing agent and was snap-frozen in isopentane for 1 min. These specimens were used for protein analysis and remained at −80 °C for a maximum of 48 h before homogenization and further handling. 

### 4.4. Histopathological Analysis 

Tissues were fixed in 4% formalin, dehydrated, embedded in paraffin wax, cut in 5 μm slices and stained with Hematoxylin-eosin (H&E) staining [39]. More specifically, tissue sections were deparaffinized, 2 × xylene incubations, 3 × ethanol incubations in 100% and 95% ethanol, followed by water wash and hematoxylin staining. After another wash, slices were differentiated using an acidic buffer, washed once with water, and incubated in 95% ethanol, followed by eosin staining for less than 1 min. 3 × washes with ethanol and 2 × washes with xylene were applied after the eosin staining, and tissue slices were then covered with Eukitt^®^ mounting medium (Sigma-Aldrich, Merck KGaA, Darmstadt, Germany) and observed under a microscope in a 40× magnification. 

Any differences between septic and sepsis-free animal samples were recorded, as well as all differences between different treatment groups. All tissue samples were evaluated regarding thickness, necrosis, and regeneration. More specifically, muscle was evaluated for fiber atrophy, e.g., reduced myosin fiber thickness, necrosis, and regeneration. 

### 4.5. Protein Analysis

Snap-frost tissues were homogenized (Heidolph Silent Cruser S, Heidolph Instruments GmbH & Co. KG, Schwabach, Germany) in 1 × PBS and homogenates were assessed for total protein concentration using the Bradford method. More specifically, homogenates were centrifuged (1000 g, 4 °C) for 20 min, and 5 μL of the supernatant was added to 200 μL BIORAD solution (Bio-Rad Laboratories, Inc, California, USA and 795 μL H_2_O. OD was measured in a photometer (595 nm). Protein concentration in the homogenates was estimated with the help of a standard curve that was made by using known concentrations of BSA. 4μg of total protein was used for dot-blot analysis [39] using the following antibodies: anti-AMPK rabbit monoclonal antibody (1:1000, Cell Signaling), anti-caspase-3 rabbit monoclonal antibody (1:1000, Cell Signaling), anti-cyclin-D1 rabbit monoclonal antibody (1:200, Abcam) and anti-β-actin mouse monoclonal antibody (1:3000, Cell Signaling) followed by ECL. In the dot-blot method, the pre-arranged amount of total protein is loaded directly on a nitrocellulose membrane through a grid, and after the protein is transferred to the membrane, blocking of non-specific binding with BSA takes place. After washing, a primary antibody and a secondary antibody linked to HRP are used. Normalization using a BSA standard curve is done, and the intensity levels of each detected protein were expressed as a protein/β-actin ratio. The use of β-actin as a loading control protein has been selected since the expression of this protein is irrelevant to any treatment and stable in all muscle samples. Before reprobing with the β-actin antibody, two consecutive 10 min-long strippings were performed. The “Gel” analysis commands of Image J software were used for protein level quantification. 

The selection of antibodies used was based on our attempt to evaluate the mechanism through which colistin mediates its effect on the muscle. Therefore, the levels of three different proteins were estimated, AMPK, caspase-3 and cyclin-D1. These proteins are involved in three different processes. AMPK is involved in cellular energy homeostasis and is activated in response to low levels of ATP in cells. Loss of AMPK activity leads to aberrant accumulation of autophagy-related proteins and defective mitophagy, thus, connecting cellular energy sensing to autophagy and mitophagy [33]. Caspase-3 is a protease enzyme that plays a central role in the process of programmed cell death, or apoptosis. It is involved in cleaving a variety of cellular proteins, leading to the characteristic morphological changes of apoptosis, including DNA fragmentation and cell shrinkage [40,41,42]. Finally, cyclin-D1 is a key regulator of the G1 phase of the cell cycle and is involved in promoting progression through the cell cycle [43].

### 4.6. Colistin Molecular Target Prediction and Docking

As mentioned above, in this study, we used the clinically available form of colistin, COLISTIN/NORMA^®^. This drug, based on the Greek National Organization for Medicines (ΕOΦ) drug catalog “Galinos” (colistimethate sodium), corresponds to the PubChem database compound CID 131704173. These data were used for colistin target prediction. Briefly, in order to gain a better understanding of the potential molecular targets of colistin in the skeletal muscles and/or neuromuscular junction of rats, we employed two freely available databases: ChEMBL and SwissTargetPrediction. These two resources provide target predictions for small molecules using different data sources, computational methods, and validation approaches. ChEMBL is a comprehensive database of bioactive compounds with annotated target information [44], while SwissTargetPrediction focuses on predicting molecular targets based on machine learning models and chemical similarity [45,46]. In the ChEMBL database, we searched for *“colistimethate sodium”* and its alternative names, while in SwissTargetPrediction, we uploaded the SMILES format of CID 131704173.

After identifying acetylcholinesterase as a potential neuromuscular junction target of colistin in rats, the PDB files of colistin and the 3D acetylcholinesterase structures obtained from UniProt (i.e., UniProt_P37136 AlfaFold predicted structure, [47,48]) were prepared for docking, following the methodology described by Bitencourt-Ferreira & de Azevedo (2019) [49,50] and uploaded to the SwissDock docking web service. The resulting docking data were visualized using the Chimera software tool for molecular visualization and analysis [51]. 

In this study, acetylcholine was chosen as a reference molecule for comparative reasons. The binding of the two molecules to acetylcholinesterase was evaluated by analyzing their respective DeltaG values. DeltaG is a thermodynamic parameter that quantifies the change in free energy associated with the binding of a ligand to a protein. It provides a measure of the stability of the ligand-protein complex and is commonly used to compare the binding affinities of different ligands. A more negative DeltaG value indicates a stronger binding affinity between a ligand and its macromolecular target.

### 4.7. Statistical Analysis

All data are expressed as means  ± SEM, and N refers to the number of independent samples. Differences between means were analyzed using the Mann–Whitney test. A comparison was considered significant when *p* < 0.05. The statistical analysis was performed using the GraphPad Prism software. The image analysis was performed using the Image J software.

## 5. Conclusions

Colistin is an antibiotic of last resort used for treating multidrug-resistant infections in critically ill patients. However, its adverse effects, including myopathy, must be closely monitored. Our study reveals that colistin treatment affects muscle size, structure, and morphology, indicating the presence of myopathy, which can be related to a possible colistin-acetylcholinesterase interaction. Moreover, we observed a lowering of AMPK concentration, induced necrosis, and inhibited cell proliferation. These effects were more pronounced in septic animals and were improved in the presence of vasoconstrictive drugs. Despite these important findings, further research is needed to fully understand the underlying mechanisms of colistin-induced myopathy and determine the potential clinical implications for critically ill patients in the ICU. Investigating these pathways could provide critical insights into how colistin exerts its effects and guide healthcare professionals in making informed decisions about its administration and dosing. Ultimately, such knowledge will improve the clinical management of critically ill patients receiving colistin and may lead to improved outcomes for this vulnerable patient population.

## Data Availability

No data.
